# Effects of Benzo[a]Pyrene Exposure on Lung Cancer: A Mechanistic Study of Epigenetic m6A Levels and YTHDF1

**DOI:** 10.3390/toxics13040280

**Published:** 2025-04-05

**Authors:** Siyi Xu, Jie Li, Sheng Yang, Panpan Yang, Yiru Niu, Yiling Ge, Geyu Liang

**Affiliations:** Key Laboratory of Environmental Medicine Engineering, Ministry of Education, School of Public Health, Southeast University, Nanjing 210009, China; 15851876530@163.com (S.X.); lijies7@163.com (J.L.); 101300318@seu.edu.cn (S.Y.); yangpanpan199912@163.com (P.Y.); niuyiru666@163.com (Y.N.); geyiling11@163.com (Y.G.)

**Keywords:** m6A, YTHDF1, benzo[a]pyrene (B[a]P), lung cancer, HBE-P35, A549, MAP3K6, miR-139-5p, TMT labeling

## Abstract

Benzo[a]pyrene, as the primary component of air pollutants, has been implicated in the pathogenesis of non-small-cell lung cancer (NSCLC). As an m6A reader that facilitates mRNA translation, YTHDF1 serves as a crucial regulator in tumor progression. Therefore, we established Benzo[a]pyrene(B[a]P)-induced bronchial epithelial malignant transformed cells (HBE-P35) to simulate the precancerous lesions of NSCLC and investigated the regulatory axis of YTHDF1 in both HBE-P35 and A549 lung cancer cells. A high level of m6A expression was detected in both HBE-P35 and A549 cells. Over-expression of YTHDF1 was observed in NSCLC tissues and correlated with poor overall survival in NSCLC patients. TMT labeling-based proteomic analysis and clinical lung tissue microarray assays demonstrated that CDK6 and MAP3K6 were positively correlated with YTHDF1 expression. MeRIP and RIP analyses revealed that YTHDF1 mediates the m6A-dependent regulation of CDK6 and MAP3K6 protein expression. The acquisition and deletion of miR-139/145-5p, along with luciferase reporter gene assays, demonstrated that miR-139-5p can target YTHDF1. Therefore, we conclude that YTHDF1 regulates CDK6 and MAP3K6 through m6A in B[a]P-induced HBE-P35 and A549 cells, providing a potential target for lung cancer treatment.

## 1. Introduction

Lung cancer ranks among the most prevalent malignancies globally. Despite recent advances in diagnostic and therapeutic strategies, lung cancer remains the second most common malignancy by incidence (11.4%) and the foremost cause of mortality (18%), according to the Global Cancer Statistics 2020 [1]. Therefore, there is a pressing need for extensive research aimed at identifying potential biomarkers for early diagnosis and developing targeted therapeutic strategies [2].

Benzo(a)pyrene (B[a]P), belonging to the polycyclic aromatic hydrocarbon family, predominantly exists in cigarette smoke, food [3], and atmospheric pollutants. The initial study of benzo(a)pyrene commenced in 1974 [4], with subsequent investigations exploring the relationship between B[a]P and the development of lung cancer [5,6,7]. Benzo(a)pyrene-trans-7,8-diol-9,10-epoxide (BPDE), a metabolite of B[a]P, is a well-known mutagen and carcinogen [8,9]. In 1996, Denissenko et al. exposed HeLa cells and human bronchial epithelial (HBE) cells to various concentrations of BPDE, finding strong adduct signals on guanine residues at positions 157,248, and 273 of the TP53 gene, indicating that different concentrations of BPDE caused mutations in the TP53 gene in both HeLa and HBE cells at these sites [10]. B[a]P contributes to lung cancer initiation, progression, and metastatic dissemination. Jiang et al. revealed that smoke can stimulate SIBLING expression and contribute to mesenchymal stem cell (MSC)-mediated lung cancer metastasis [11]. While B[a]P exposure significantly correlates with lung cancer initiation and progression, the precise molecular mechanisms and signaling cascades have yet to be fully elucidated.

Epigenetic modifications are closely associated with tumorigenesis and progression [12]. Methylation modifications of RNA are a form of epigenetic regulation that post-transcriptionally modulate gene expression without changing the underlying nucleotide sequence. M6A modification, occurring at adenine (A) residues, represents the most abundant RNA epigenetic mark across eukaryotic species [13,14,15]. Additionally, the presence of m6A modifications, which can be found across virtually all coding and non-coding RNA species, plays a dynamic role in modulating their respective molecular processes [16]. The dysregulation of m6A regulatory mechanisms is associated with tumorigenesis and progression [17,18]. YTH domain family 1 (YTHDF1), an m6A “reader”, is expressed at elevated levels in various cancers [19,20,21,22]. Numerous studies have highlighted the pivotal role of YTHDF1 in tumorigenesis. As an m6A reader protein, YTHDF1 has been shown to promote hepatocellular carcinoma cell growth, motility, invasiveness, and cell cycle progression by activating the PI3K/AKT/mTOR signaling pathway [23]. Studies have revealed that YTHDF1 expression correlates with disease advancement in hypopharyngeal squamous cell carcinoma [24]. Additionally, YTHDF1 may promote gastric carcinogenesis by regulating the translation of FDZ7 [25]. The exact molecular mechanisms through which YTHDF1, acting as an m6A reader, regulates lung cancer initiation and progression have yet to be fully elucidated. Therefore, investigating the regulatory mechanisms of YTHDF1 in lung cancer is clinically significant.

This research explored how YTHDF1 regulates both malignantly transformed bronchial epithelial cells and A549 lung cancer cells. We first established B[a]P-induced bronchial epithelial malignant transformed cells (HBE-P35), detected the changes in YTHDF1 and m6A levels in lung cancer and malignant transformed cells, and then used proteomics, MeRIP, and RIP to probe the upstream and downstream regulatory proteins of YTHDF1. We expected to find potential therapeutic targets for lung cancer through the above studies.

## 2. Materials and Methods

### 2.1. Datasets

Tissue mRNA and miRNA sequencing data from a total of 521 LUAD patients and 504 LUSC patients was obtained from the TCGA database (http://cancergenome.nih.gov/publications/publicationguidelines/, accessed on 12 February 2017), along with their basic information. Following the application of this study’s inclusion and exclusion criteria, the final analysis incorporated data from 465 LUAD patients and 429 LUSC patients. The mRNA and miRNA sequencing data in the TCGA database were level 3 visual data, which could be analyzed directly without the need for normalization or standardization. The results of all publicly available sequencing data analyses and survival analyses for LUAD in the TCGA and GEO databases were obtained from the LUADEXPRESS website (http://www.bioinfo-zs.com/luadexpress/, accessed on 15 March 2021).

### 2.2. Tissue Microarray and Immunohistochemistry Analysis

Tissue microarrays were sourced from Aifang Biotechnology Company (Changsha, China). These microarrays comprised 160 lung tissue samples. Following standard pathological protocols, the specimens underwent sequential processing, including 4% paraformaldehyde fixation, pruning, dehydration, embedding, sectioning, and staining, before microscopic examination and sealing.

### 2.3. Reverse-Transcription Quantitative (RT-q)PCR

RNA was extracted using Trizol Reagent (Invitrogen, Carlsbad, CA, USA) following the manufacturer’s protocol. RNA was then reverse transcribed using HiScript-II Q RT SuperMix (Vazyme, Nanjing, China), followed by quantitative PCR analysis with 2 × ChamQ SYBR Color qPCR Master Mix. The primers used for gene amplification are listed in Table S1.

### 2.4. Constructing Bronchial Epithelial Malignant Transformed Cells HBE-P35

B[a]P was initially dissolved in DMSO to prepare a stock solution at a concentration of 40 μmol/mL. This stock was then diluted with RPMI-1640 culture medium to achieve a final concentration of 80 μmol/L. For B[a]P exposure, HBE cells at 80% confluence were exposed to B[a]P (80 μmol/L) or the solvent control (DMSO) for 24 h, once per passage. Next, the medium was replaced with standard RPMI-1640 complete medium, and the cells were cultured until they reached the next passage, with this process being repeated 35 times, up to passage 35 (P35). P35 cells served as the experimental cells for this study and were designated as HBE-P35, while normally cultured 16HBE cells were used as the normal control and designated as HBE. The HBE cells (CVCL_3695) we used were purchased from the Typical Culture Preservation Committee cell bank of the Chinese Academy of Sciences, and the A549 cells (CVCL_0023) were purchased from the Typical Culture Preservation Committee cell bank of the Chinese Academy of Sciences.

### 2.5. Cell Cycle Assay

The cells were washed with PBS and then digested with trypsin. Following the PBS wash and centrifugation, the supernatant was discarded. Next, 500 μL of 70% ethanol was added to 1 × 10^6^ cells at 4 °C to fix the cells overnight. The fixed cells were washed with PBS to remove the fixative, then centrifuged again as previously described. Following this, 500 μL of PI/RNase A staining solution (9:1 ratio) was added to each 1 × 10^6^ cells. The cells were incubated at room temperature, protected from light, for 30 to 60 min before being analyzed using a flow cytometer (Thermo, Waltham, MI, USA). Red fluorescence was detected at an excitation wavelength of 488 nm. Three parallel samples were prepared for each cell type, and each sample was analyzed in triplicate with the flow cytometer.

### 2.6. Cell Transfection

Nanjing Corex Biologicals (Nanjing, China) provided the sh-YTHDF1 lentiviral vector and its negative control, utilizing lentiviral interfering shuttle vectors with puromycin (Puro) resistance and GFP tags. The lentiviral sequence and titer information are presented in Table S1. Cells were plated in 6-well plates at a density of 3 × 10^5^ cells per well, and lentivirus was transduced at a multiplicity of infection (MOI) of 30, corresponding to 50–60% cell confluence. An appropriate amount of polybrene was added to each well, gently mixed, and then the plates were placed in the incubator. The cells were gently washed with PBS, and fresh medium was added. They were then incubated for another 48 h, until fluorescence was observed in the cells. Successfully transduced cells were maintained in complete RPMI-1640 containing puromycin. A high concentration of puromycin (8–10 µg/mL) may be used at the onset of the screening, and as the screening progresses, the concentration is gradually reduced to 2 µg/mL and then maintained for continued culture.

Three siRNA sequences were designed for the transient knockdown of YTHDF1 expression (refer to Table S1 for sequence details). Cells were collected and plated into the appropriate well plates 12 h before transfection, reaching approximately 50% confluence. The siRNA sequences were delivered into the cells using the riboFECT CP Transfection Kit (Guangzhou Ruibo biological Technology Co., Ltd., Guangzhou, China), in a medium without penicillin–streptomycin or serum. Gene expression was observed between 24 and 72 h post transfection.

The miRNA-139/145-5p mimic and inhibitor were provided by Reebok Bio (Guangzhou, China), and the sequences of the mimic and inhibitor are kept confidential by the company. The transfection method utilized is identical to that of YTHDF1.

Six siRNA sequences were designed for the transient knockdown of CDK6 and MAP3K6 expression (refer to Table S1 for sequence details). The transfection method utilized is identical to that of YTHDF1.

### 2.7. Proteomic Sample Preparation Followed by LC-MS/MS

The proteins were labeled according to the protocol provided with Thermo’s TMT labeling kit (Thermo, Waltham, MI, USA). Reversed-phase (RP) and strong cation exchange (SCX) fractionation were performed on equally mixed labeled peptides from each group. The flow rate of the HPLC system was 300 nL/min. Buffer A (0.1% formic acid aqueous) and Buffer B (0.1% formic acid acetonitrile) were used. The linear elution gradient was as follows: 6% Buffer B for 5 min, 6–30% Buffer B for 60 min, 30–40% Buffer B for 10 min, 40–100% Buffer B for 5 min, and 100% buffer B for 5 min. Separation was performed using a Thermo Scientific EASY column. The analysis was carried out using a Q-Exactive mass spectrometer (Thermo Fisher Scientific, Waltham, MI, USA). The parent ion scanning range was 300–1800 *m*/*z*, the primary mass spectrometry resolution was 70,000 (200 *m*/*z*), the AGC (automatic gain control) was 10^6^, and the maximum IT was 50 ms. The dynamic exclusion time was 60.0 s. The mass charge ratio was collected according to the following methods: After each full scan, 20 fragments were collected. The HCD isolation window was 2 *m*/*z*, the secondary mass spectrometry resolution was 17,500 (200 *m*/*z*), and the normalized collision energy was 30 eV. Underfill was 0.1%. For TMT quantitative proteomic sequencing, differentially expressed proteins were identified based on fold changes (FC) > 1.2 or FC < 0.83 and *p* < 0.05.

### 2.8. Cell Proliferation

Cell viability was assessed using the CCK8 (Cell Counting Kit-8, CELLCOOK, Guangzhou, China) assay. HBE, HBE-P35, and A549 cells were plated into a 96-well plate at a density of 2000–3000 cells per well. The CCK8 reagent was then added to the wells and incubated at 37 °C for 2 h. Optical density (OD) values were recorded at a wavelength of 450 nm using a Multiskan SkyHigh (Thermo Fisher Scientific, USA).

### 2.9. Luciferase Reporter Assay

Luciferase reporter gene assays were performed using the Dual-Luciferase Reporter Assay System (Yeasen, Shanghai, China), following the manufacturer’s guidelines.

### 2.10. Determination of Total m6A

Experiments were performed using the EpiQuikTM m6A RNA Methylation Quantification Kit (Amyjet, Wuhan, China).

### 2.11. Methylated RNA Immunoprecipitation Sequencing and RIP Assay

The EZ-Magna RIP RNA-Binding Protein Immunoprecipitation Kit (Merck, Darmstadt, Germany) was utilized to perform the assay. First, cells were lysed using a custom-formulated RIP lysis buffer, and the cell extracts were subsequently incubated with IgG and antibodies at 4 °C overnight. RNA was purified from the complexes, and cDNA was synthesized before being subjected to RT-qPCR.

### 2.12. Transwell Assay

For cell migration assays, cells were plated into the upper chamber. In total, 600 μL of medium supplemented with 20% fetal bovine serum was added to the lower chamber. Cells were fixed with paraformaldehyde and stained with violet after 24 h. Samples were examined under a microscope, and a field of view was selected for cell counting. For the invasion assay, 50 μL of a matrigel and serum-free medium mixture (1:8 ratio) was added to the upper chamber and incubated at 37 °C for 30 min. The remaining steps are identical to those of the cell migration assays.

### 2.13. Wound Healing Assay

A single layer of cells was scratched with the tip of a sterile 1 mL pipette to simulate a wound. Two milliliters of serum-free medium were added, and the cells were incubated. The wound was examined and analyzed under a microscope after incubation for 0, 6, 12, 24, 48, and 72 h. The scratch area was quantified using the ImageJ software (https://ij.imjoy.io/ accessed on 21 March 2023), and the healing ability of the cells was subsequently analyzed.

### 2.14. Western Blot Analysis

The gel was prepared by mixing liquid A and liquid B of the PAGE gel in a 1:1 ratio, and then equivalent quantities of total protein were subjected to electrophoretic separation. The separated proteins underwent electrotransfer to a PVDF membrane, followed by a single TBST rinse. The membrane was then subjected to a blocking step using 5% skim milk in TBST for 2 h. Following blocking, the PVDF membrane was incubated with primary antibody overnight at 4 °C. Subsequently, three 15 min washes with TBST were performed at room temperature. The PVDF membrane was incubated with the secondary antibody for 1 h, washed with TBST 3 times for 15 min, and then the chemiluminescence assay was performed using ECL substrate (Amersham Biosciences, Amersham, UK). The gray values were quantified using ImageJ for statistical data analysis.

### 2.15. Statistical Analysis

SPSS 26.0 software was used to conduct statistical analyses. Significant differences are denoted by asterisks (*) in the figures. Statistical comparisons between two groups were conducted using independent samples *t*-tests, and one-way ANOVA was utilized for multi-group data analysis. A statistical comparison of survival between the two groups was conducted using the Log-rank test, with statistical significance defined at *p* < 0.05. Statistical graphs were created using GraphPad Prism 7.0.

## 3. Results

### 3.1. Constructing HBE Cell Malignant Transformation Model Through B[a]P Exposure

**Figure 1:**
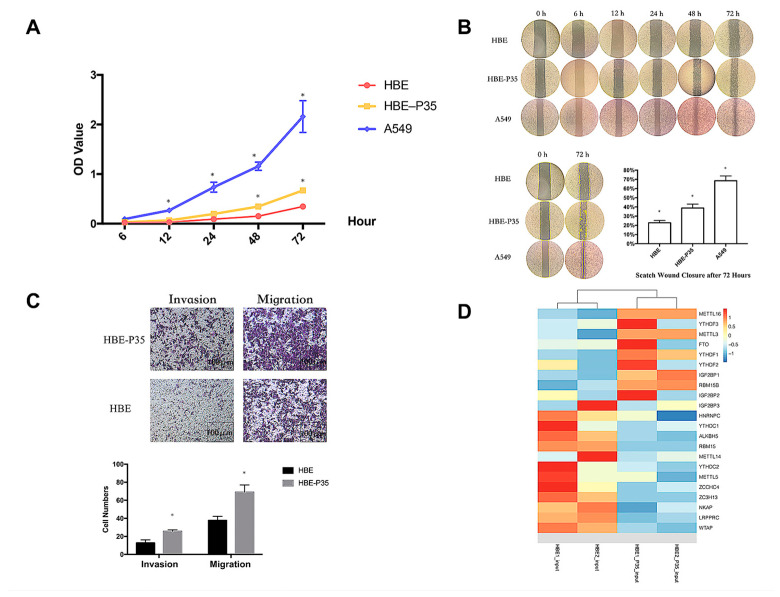
Long-term B[a]P exposure leads to a malignant phenotype in HBE cells. (**A**) Long-term B[a]P exposure enhances the proliferative capacity of HBE-P35 cells; (**B**) scratch assay of HBE and A549 cells with long-term B[a]P exposure; (**C**) long-term B[a]P exposure promoted HBE-P35 cell invasion and metastasis; (**D**) expression of m6A-related genes in transcriptome sequencing results of HBE and HBE-P35 cells. Values are presented as the mean ± SD. * *p* < 0.05, compared with passage-matched control HBE cells.

The malignant phenotype of HBE-P35 cells was characterized by evaluating their proliferative capacity and metastatic potential through migration and invasion assays. The results demonstrated that after 35 generations of chronic exposure to the chemical B[a]P, the proliferative capacity of HBE-P35 gradually increased compared to normal bronchial epithelial cells (HBE) (Figure 1A). Cells chronically exposed to B[a]P demonstrated a stronger wound healing ability compared to unexposed HBE cells (Figure 1B). The results of the cell invasion and metastasis experiments further demonstrated that HBE cells exhibited a malignant phenotype following prolonged exposure to B[a]P (Figure 1C). These findings imply that B[a]P can induce the malignant transformation of HBE cells and promote tumor development in vitro.

### 3.2. m6A Modification Is Implicated in the Malignant Transformation Process Induced by B[a]P Exposure

Transcriptome sequencing and MeRIP sequencing were employed to detect transcriptional and epigenetic changes in the malignant transformed HBE-P35 cells. To determine whether the level of m6A modification changes during lung cancer development, the total expression levels of m6A modification in HBE-P35 cells and lung cancer cells were examined. The results revealed that the level of m6A modification was elevated in HBE-P35 and lung adenocarcinoma cell line A549 compared to normal HBE cells (Figure S1A). To understand the impact of B[a]P exposure on the transcriptome and gene expression of HBE cells, next-generation sequencing (NGS) transcriptome sequencing was conducted on HBE and HBE-P35 cells. The results indicated that after continuous exposure to B[a]P, there are many differentially expressed transcripts and genes in HBE-P35 cells (Figure S1B–D). GO and KEGG enrichment analyses indicated that B[a]P exposure activated numerous classical pathways closely related to cancer occurrence and development, such as the PI3K signaling pathway, p53 signaling pathway, and ECM receptor (Figure S2A,B). Further analysis of the expression of m6A-related genes indicated that YTHDF1 was upregulated in HBE-P35 cells, suggesting that B[a]P exposure could promote the level of m6A modification and related protein expression to a certain extent (Figure 1D). MeRIP-Seq results indicated that there are lots of m6A-modified genes affected by B[a]P exposure (Figure S1E,F). This is not only fundamentally consistent with the results of whole-transcriptome sequencing but also indicates that m6A modification is indeed affected by B[a]P exposure and is involved in the carcinogenic and promoting processes associated with B[a]P.

### 3.3. YTHDF1 Regulates the Proliferation, Migration, and Invasion

**Figure 2:**
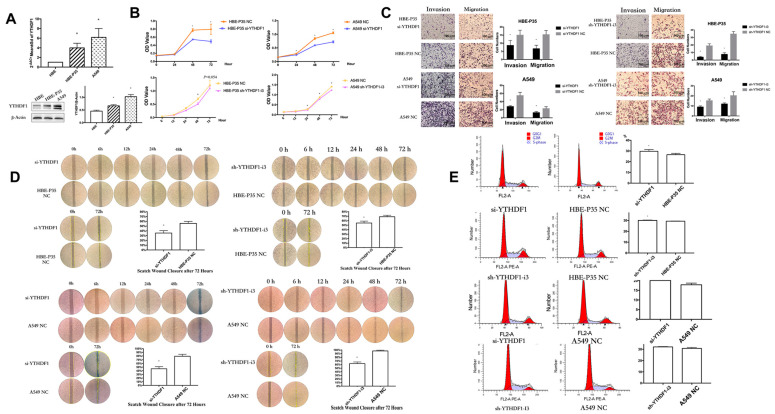
Knocking down YTHDF1 inhibits the growth, migration, and wound healing ability of HBE-P35 and A549 cells. (**A**) qPCR and WB analysis for YTHDF1 in HBE, HBE-P35, and A549. (**B**) CCK8 assays in A549 and HBE-P35 cells. (**C**) Transwell experiments showed that knocking down YTHDF1 reduced the invasion and migration ability of A549 cells and HBE-P35 cells. (**D**) Wound healing experiments showed that a low expression of YTHDF1 reduced the wound migration ability of cells. (**E**) Cell cycle experiments showed that the knockdown of YTHDF1 caused S-phase arrest of cells. Data are shown as means ± S.D.; * *p*  <  0.05.

YTHDF1 expression may be linked to the prognosis of lung cancer, as indicated by data from the TCGA and GEO databases (Figure S3). PCR and Western blot experiments on HBE, HBE-P35, and A549 cells demonstrated that the expression of YTHDF1 was higher in malignant transformed and lung cancer cells compared to HBE cells (Figure 2A). siRNAs and shRNAs targeting YTHDF1 were utilized to knock down YTHDF1 expression in HBE-P35 and A549 cells (Table S1). Subsequently, the cell assay confirmed that the viability of HBE-P35 and A549 cells with YTHDF1 knockdown was inhibited (Figure 2B). The migration and invasion capabilities of YTHDF1 was inhibited (Figure 2C). The wound healing further indicated that YTHDF1 promoted the migration of HBE-P35 and A549 cells (Figure 2D). Cell cycle experiments indicated that YTHDF1 knockdown caused S-phase blockade in HBE-P35 and A549 cells (Figure 2E). Collectively, the above functional experiments demonstrate that YTHDF1 regulates the proliferation, migration, and invasion of lung cancer cells. Therefore, a comprehensive investigation of YTHDF1 is essential for understanding the pathogenesis of the disease.

### 3.4. YTHDF1 Regulates Lung Carcinogenesis and Progression by Modulating CDK6 and MAP3K6

TMT labeling-based proteomic analysis of HBE-P35 and HBE-P35 cells was conducted with YTHDF1 knocked out (Figure S4). MAP3K6, CDK6, and PARD6β were found to be not only downregulated in YTHDF1 knockdown cells (Table S2) but also associated with cell cycle (Table S3). Therefore, we focused on these three proteins in our subsequent studies.

**Figure 3:**
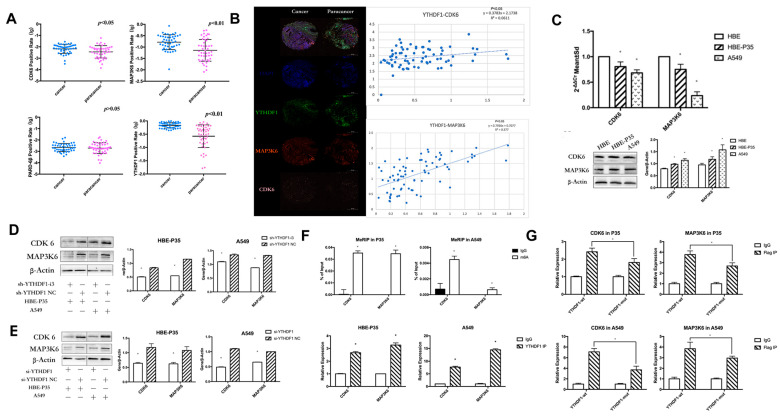
YTHDF1 regulates CDK6 and MAP3K6 mRNA expression through m6A. (**A**) The expression of YTHDF1, CDK6, MAP3K6, and PARD-6β in clinical lung cancer tissues by immunohistochemical staining (IHC) using tissue microarrays. (**B**) Relationship between CDK6 and MAP3K6 as well as YTHDF1 in clinical lung cancer tissues. (**C**) qPCR and WB of CDK6 and MAP3K6 in A549, HBE, and HBE-P35 cells. (**D**,**E**) After YTHDF1 knockdown, CDK6 and MAP3K6 protein expression levels. (**F**) Top: m6A Me-RIP followed by RT-qPCR. Bottom, YTHDF1 RIP followed by RT-qPCR. (**G**) RT-qPCR in A549 and HBE-P35 cells. Data are shown as means ± S.D.; * *p*  <  0.05.

YTHDF1 regulates the expression levels of CDK6 and MAP3K6 proteins through m6A modification. We examined the expression of YTHDF1, CDK6, MAP3K6, and PARD-6β in clinical tissues using immunohistochemical staining (IHC) with tissue microarrays. The experimental data indicated that YTHDF1, MAP3K6, and CDK6 were significantly over-expressed in lung cancer compared to adjacent normal tissues (*p* < 0.05) (Figure 3A). Additionally, a positive correlation was observed between the expression levels of YTHDF1, MAP3K6, and CDK6 (Figure 3B). Moreover, the analysis revealed that CDK6 expression could significantly distinguish between LUAD and LUSC patients (Table S4). Unfortunately, no clear difference in PARD-6β expression was observed between paracancerous and lung cancer tissues. Moreover, no notable correlation was found between the expression levels of YTHDF1 and PARD-6β. Therefore, subsequent studies targeting downstream regulatory proteins of YTHDF1 will primarily focus on CDK6 and MAP3K6. Furthermore, the expression of CDK6 and MAP3K6 in HBE, HBE-P35, and A549 cells indicated that protein levels were significantly elevated in A549 and HBE-P35 compared to HBE (*p* < 0.05) (Figure 3C). This indicates that the expression of CDK6 and MAP3K6 was elevated during the transformation of normal cells into lung cancer cells. We further found that the expression of CDK6 and MAP3K6 proteins was also decreased in cells that knocked down YTHDF1, indicating a certain positive correlation between YTRHDF1 and CDK6 and MAP3K6 (Figure 3D,E). We subsequently examined the m6A modification status of CDK6 mRNA and MAP3K6 mRNA in HBE-P35 and A549 cells using a MeRIP-qPCR assay, confirming the presence of m6A modification sites on both CDK6 and MAP3K6 mRNA; furthermore, both were shown to bind to the YTHDF1 protein (Figure 3F). To investigate whether YTHDF1 regulates CDK6 and MAP3K6 through m6A-dependent regulation (Figure S5E), HBE-P35 and A549 cells were transfected with YTHDF1-mut (YTHDF1 with FLAG tagging in the YTH structural domain, introducing the K395A and Y397A mutation sites) and YTHDF1-wt (wild-type YTHDF1). Subsequent RIP-qPCR experiments validated the interaction between YTHDF1 and both CDK6 and MAP3K6 in HBE-P35 and A549 cells (Figure 3G). RIP analysis using an anti-FLAG antibody followed by quantitative PCR (qPCR) demonstrated efficient immunoprecipitation of CDK6 and MAP3K6 mRNA in cells transfected with YTHDF1-wt. In contrast, cells transfected with the YTHDF1-mut variant showed the significantly reduced immunoprecipitation of both CDK6 and MAP3K6 mRNA. This suggests that YTHDF1 regulates CDK6 and MAP3K6 mRNA through m6A modification.

### 3.5. CDK6 and MAP3K6 Regulate Proliferation, Cell Cycle, and Invasion

**Figure 4:**
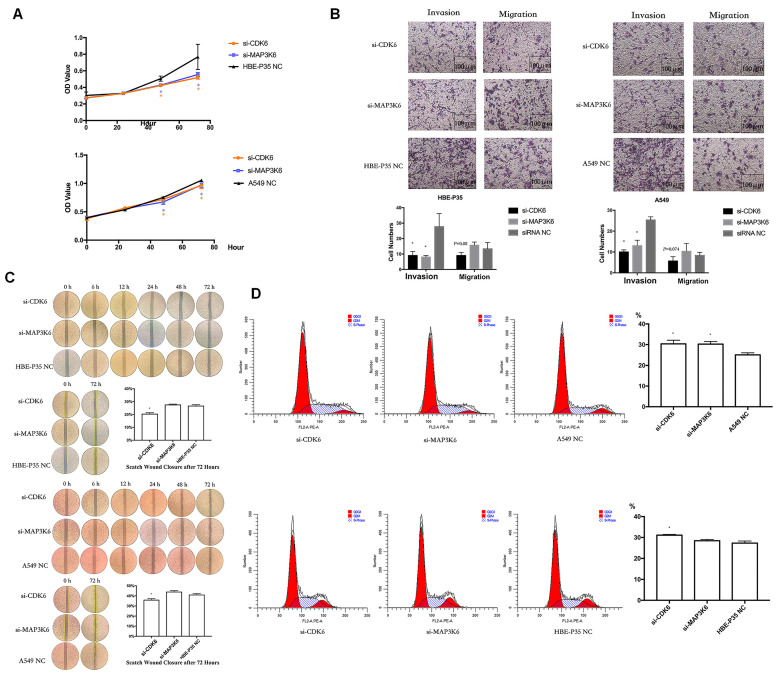
(**A**) CCK8 assays in A549 and HBE-P35 cells. (**B**) Transwell experiments showed that knocking down CDK6 and MAP3K6 reduced the invasion ability of A549 cells and HBE-P35 cells. (**C**) Wound healing experiments showed that a low expression of CDK6 reduced the wound migration ability of cells. (**D**) Cell cycle experiments showed that the knockdown of CDK6 caused S-phase arrest of A549cells and HBE-P35 cells. Data are shown as means ± S.D.; * *p*  <  0.05.

Cell growth, wound healing, cell cycle progression, migration, and invasion were carried out to explore the roles of CDK6 and MAP3K6. The results indicated that A549 and HBE-P35 cells with CDK6 knockdown exhibited inhibited growth (Figure 4A), wound healing (Figure 4C), and invasion (Figure 4B). Notably, S-phase blockade occurred in cells with CDK6 knockdown (Figure 4D). However, CDK6 knockdown had a limited effect on the number of cell migrations across the membrane, suggesting that CDK6 protein still possesses the ability to promote cell migration. As a cyclin, migration may not be the primary cellular function of CDK6. The knockdown of MAP3K6 inhibited the proliferation and invasion of both HBE-P35 and A549 cells (Figure 4A,B). MAP3K6 knockdown had no significant effect on wound healing and cell migration, indicating that MAP3K6 does not promote cell migration (Figure 4B,C). The S-phase arrest observed in A549 cells, but not in HBE-P35 cells, was attributed to MAP3K6 knockdown, possibly because HBE-P35 cells did not undergo sufficient malignant transformation, and MAP3K6 primarily promoted the cell cycle in cancer cells (Figure 4D). In summary, these results suggest that CDK6 and MAP3K6 function to promote lung carcinogenesis.

### 3.6. miR-139/145-5p Are Upstream Regulators of YTHDF1 in the Progression of Lung Cancer

Differential analysis of paired lung cancer tissue samples from TCGA revealed that the expression levels of 122 miRNAs were significantly regulated in lung cancer tissues (*p* < 0.05, |log2FC| > 1). Among these, 34 miRNAs were significantly under-expressed, while 88 miRNAs were significantly over-expressed (Figure S6A,B). The miRWalk and starBase databases were further applied to predict the upstream miRNAs of YTHDF1, while intersections were taken with the above differential miRNAs. The results showed that among the 11 miRNAs in the intersection, miR-184, miR-139-5p, and miR-145-5p were under-expressed in lung cancer tissues, which were inversely proportional to the expression of YTHDF1 and might be the key miRNAs regulating YTHDF1 (Figure S6C). Analysis of the GEO database showed that only miR-139-5p and miR-145-5p were specifically under-expressed in lung cancer patients (*p* < 0.05, |log2FC| > 1), and the expression of miR-184 was inconsistent and did not match the predicted results (Table S5). PCR reveals the expression of hsa-miR-139/145-5p decreased in HBE-P35 cells, and A549 cells compared to HBE cells (Figure S6D). Therefore, we now focus on the mechanism of miR-139-5p and miR-145-5p in lung cancer.

**Figure 5:**
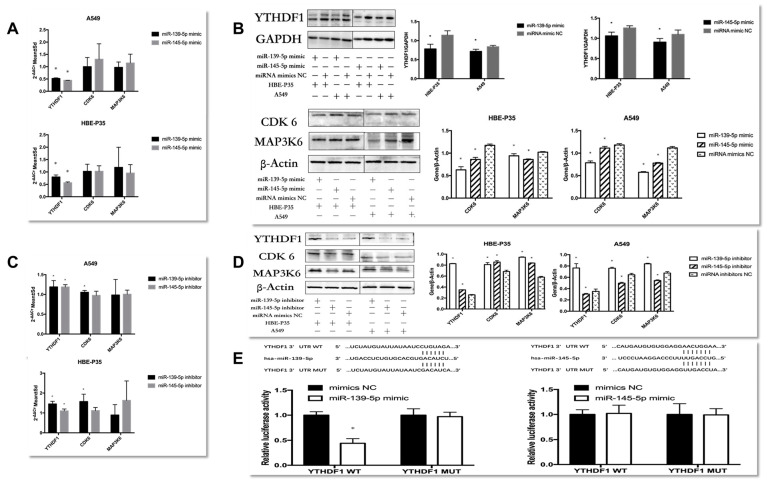
Explore the regulatory role between YTHDF1 and hsa-miR-139/145-5p. (**A**,**B**) RT-qPCR and Western blot explore the expression of YTHDF1, CDK6, and MAP3K6 in hsa-miR-139/145-5p over-expressed in A549 cells and HBE-P3 cells. (**C**,**D**) The expression of YTHDF1, CDK6, and MAP3K6 in A549 cells and HBE-P35 cells with low expression of hsa-miR-139/145-5p was detected by RT-qPCR and Western blot. (**E**) Luciferase reporter genes reveal that miR-139 could reduce cellular luciferase activity. Data are shown as means ± S.D.; * *p*  <  0.05.

The regulatory relationships between miRNAs and YTHDF1 were investigated. First, the experimental results indicated that the expression of YTHDF1 was downregulated in HBE-P35 and A549 cells over-expressing miR-139/145-5p (Figure 5A,B). The proteins levels of CDK6 and MAP3K6 changed significantly, while the mRNA levels did not change (Figure 5A,B). These results showed that miR-139-5p/miR-145-5p were negatively correlated withYTHDF1 expression. Furthermore, the regulatory relationship between miR-139-5p/miR-145-5p and the downstream genes CDK6 and MAP3K6 mirrored the effect of YTHDF1 on these two genes.

When HBE-P35 and A549 cells were treated with miR-139-5p inhibitors (Figure 5C,D), both YTHDF1 mRNA and protein expression were elevated with statistical significance in HBE-P35 and A549 cells (*p* < 0.05). The protein expression of CDK6 and MAP3K6 was also elevated; conversely, the reduction in miR-139-5p expression promoted CDK6 mRNA elevation and had no effect on MAP3K6 mRNA. In HBE-P35, when miR-145-5p expression was suppressed, it still had a negative regulatory relationship on the expression of YTHDF1, CDK6, and MAP3K6 protein and had no effect on CDK6 and MAP3K6 mRNA. However, it showed the inhibition of the proteins of YTHDF1, CDK6, and MAP3K6 in A549 cells. In conclusion, miR-139-5p was negatively correlated with YTHDF1 expression in both HBE-P35 and A549 cells, and it increased the protein expression of both CDK6 and MAP3K6. In this regulatory process, the inhibition of miR-139-5p expression exerted a negative regulatory effect on CDK6 mRNA. This finding suggests that miR-139-5p, as an upstream regulator, may modulate CDK6 through multiple regulatory axes and mechanisms. While the regulatory relationship of miR-145-5p on YTHDF1, CDK6, and MAP3K6 showed diametrically opposite results in HBE-P35 and A549 cells, which needs to be supplemented by subsequent experiments.

To verify whether hsa-miR-139/145-5p directly targets YTHDF1 in HBE-P35 and A549 cells, we cloned YTHDF1 mRNA (designated as YTHDF1 WT) along with two mutants (designated as YTHDF1 MUT). After the co-transfection of YTHDF1 wild-type/mutant plasmids with miR-139-5p NC/mimic, only the fluorescence intensity of PmirGLO YTHDF1-3′UTR-WT in the miR-139-5p mimic group was significantly lower compared to that of the other three groups (Figure 5E). This result indicates an miR-139-5p-YTHDF1 mRNA binding interaction, with the binding site being the one predicted with TargetScan (http://www.targetscan.org/); after the co-transfection of YTHDF1 wild-type/mutant plasmid and miR-145-5p NC/mimic, there was no difference in the fluorescence intensity of all four groups (Figure 5E), indicating that miR-145-5p and YTHDF1 mRNA do not bind through the binding site predicted by TargetScan and do not bind to each other. These results indicate that YTHDF1 mRNA is a target of miR-139-5p and is directly regulated by it. By contrast, miR-145-5p does not appear to directly regulate YTHDF1.

### 3.7. miR-139/145-5P Regulate the Proliferation, Cell Cycle, Migration, and Invasion of Lung Cancer Cells

**Figure 6:**
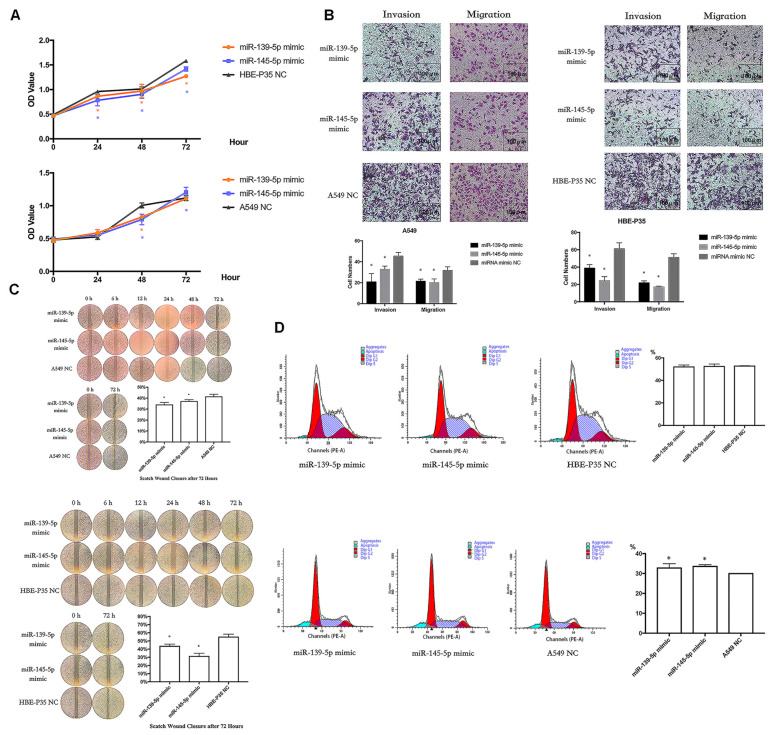
miR-139/145-5P regulates the proliferation, cell cycle, migration, and invasion of lung cancer cells. (**A**) CCK8 assays in A549 and HBE-P35 cells with hsa-miR-139/145-5p over-expressed. (**B**) Transwell experiments showed that over-expression of hsa-miR-139/145-5p inhibited the invasion and migration of cells. (**C**) Wound healing experiments showed that over-expression of hsa-miR-139/145-5p inhibited cell migration. (**D**) Cell cycle experiments showed that over-expression of hsa-miR-139/145-5p caused S-phase arrest of A549 cells. Data are shown as means ± SD; * *p*  <  0.05.

To analyze the biological function of hsa-miR-139/145-5p, two types of mimics were used to enhance the expression of hsa-miR-139/145-5p in A549 and HBE-P35 cells (Figure S5). CCK8 assays indicated that the proliferation of HBE-P35 and A549 cells was inhibited with an enhanced expression of hsa-miR-139/145-5p (Figure 6A). Transwell assays further demonstrated that the enhanced expression of hsa-miR-139/145-5p inhibited the migration and invasion capabilities of HBE-P35 and A549 cells (Figure 6B). The wound healing assay further demonstrated that hsa-miR-139/145-5p inhibited the migration of lung cancer cells (Figure 6C). Cell cycle experiments indicated that the knockdown of miR-139/145-5p in A549 cells resulted in S-phase arrest, suggesting that these two miRNAs promote cell cycle progression (Figure 6D). However, the knockdown of miR-139/145-5p in HBE-P35 cells did not lead to S-phase arrest, possibly because the malignancy of HBE-P35 cells is lower than that of lung cancer A549 cells. These results suggest that hsa-miR-139/145-5p plays a role in the development of lung cancer.

## 4. Discussion

An altered expression of m6A is strongly linked to the development of various tumors [26]. Our study showed that m6A expression was elevated in B[a]P-induced HBE-P35 and A549 cells compared to HBE cells. Tumor growth and invasion are regulated by m6A-associated genes, including “writers”, “readers”, and “erasers”, highlighting the importance of exploring the expression and interactions between m6A and its associated genes in tumors [27,28]. Although some studies have investigated the pathogenic mechanism of YTHDF1 in lung cancer [29,30,31], the progression of lung cancer results from the mutual regulation of multiple factors, with fewer studies conducted to investigate the regulatory axis of YTHDF1. Our study revealed that the YTHDF1 gene was upregulated in NSCLC within the TCGA cohorts. Subsequent experiments involving the immunohistochemical staining of clinical lung cancer tissue microarrays and detection of YTHDF1 expression levels in HBE-P35 and A549 cells further confirmed this trend. Notably, analysis of GEO survival data revealed that elevated YTHDF1 expression was associated with overall survival, suggesting that YTHDF1 mRNA expression could serve as a prognostic marker for overall survival in NSCLC. The loss-of-function experiment results showed that YTHDF1 in HBE-P35 and A549 cells promoted cell growth, wound healing, and the invasion and migration of cells. Further cell cycle experiments indicated that S-phase arrest occurred in YTHDF1 knockdown cells, suggesting that YTHDF1 functions to drive the cell cycle forward. Thus, YTHDF1 plays a crucial role in the onset and progression of NSCLC.

YTHDF1 regulates the expression of CDK6 and MAP3K6 proteins through m6A modification. To further investigate the mechanism of YTHDF1 in lung cancer, TMT labeling-based proteomic analysis was performed using cells with YTHDF1 knockdown. The results revealed a reduction in CDK6 and MAP3K6 in YTHDF1 knockdown cells. Previous studies have demonstrated that CDK6 promotes lung cancer cell proliferation by advancing the cell cycle, and that CDK6 expression is linked to overall survival in lung cancer patients [32,33]. The MAP3K family is also implicated in cancer progression [34]. Therefore, by combining TMT labeling-based proteomic analysis with previous findings, we explored the roles of CDK6 and MAP3K6 in lung cancer development and the relationship between YTHDF1, CDK6, and MAP3K6. Immunohistochemical staining (IHC) experiments on clinical lung cancer tissues using tissue microarrays demonstrated a positive correlation between YTHDF1 and its downstream proteins, CDK6 and MAP3K6. MeRIP-qPCR experiments indicated that the m6A expression of CDK6 and MAP3K6 were elevated in HBE-P35 and A549 cells. Two subsequent RIP-qPCR experiments further indicated that YTHDF1 regulates the expression levels of CDK6 and MAP3K6 proteins through m6A-dependent regulation. Our experiments also indicated that CDK6 and MAP3K6 are involved in regulating cancer cell proliferation, invasion, migration, wound healing, and cell cycle progression.

miR-139/145-5p act as upstream regulators of YTHDF1 in the development of lung cancer. A recent study suggested that environmental exposures may affect the health of the organism by influencing the expression of non-coding RNAs [35]. Analysis of the TCGA database, online resources, and our cellular experiments revealed that miR-139-5p was lowly expressed in HBE-P35 and A549 cells. Moreover, it has been shown that miR-139-5p inhibits the development of lung cancer [36,37]. Our experimental results were consistent with these findings. Furthermore, it has been shown that miR-145-5p regulates tumor development [38,39]. Our analysis also revealed that miR-145-5p was lowly expressed in lung cancer tissues and cells. Therefore, studying the function of hsa-miR-139/145-5p in non-small-cell lung cancer (NSCLC) is important.

miR-139/145-5p tended to decrease the protein levels of YTHDF1 in HBE-P35 and A549 cells. An opposite trend of YTHDF1 was observed in HBE-P35 cells with suppressed miR-139/145-5p expression. However, in A549 cells, as miR-145-5p expression decreased, YTHDF1 also exhibited a trend of downregulation, suggesting a more complex regulatory mechanism between miR-145-5p and YTHDF1 in NSCLC. The luciferase reporter assay further demonstrated that YTHDF1 mRNA was directly regulated by miR-139-5p but not by miR-145-5p. Our experimental results are consistent with those of a previous report, which indicated that miR-145-5p could directly target YTHDF2 [38]. Cellular function experiments demonstrated that miR-139-5p regulates cell proliferation, wound healing, invasion, and migration, and drives the cell cycle. In conclusion, miR-139-5p promotes lung cancer cell growth through the direct regulation of YTHDF1 mRNA.

In conclusion, B[a]P exposure can facilitate the progression of non-small-cell lung cancer (NSCLC) by altering m6A expression. miR-139-5p promotes the growth of HBE-P35 and A549 cells through the direct regulation of YTHDF1 mRNA. YTHDF1 regulates CDK6 and MAP3K6 expression through m6A in B[a]P-induced HBE-P35 and A549 cells. Since our study lacks epidemiological studies on specific populations, as well as in vivo animal studies, we will later study the toxic effects of BaP through m6A in more depth from these two aspects. In conclusion, our study presents a potential target for the diagnosis and treatment of lung cancer.

## Data Availability

The datasets generated and analyzed during the current study are available from the TCGA database (http://cancergenome.nih.gov/publications/publicationguidelines/, accessed on 12 February 2017) and GEO databases (http://www.bioinfo-zs.com/luadexpress/, accessed on 15 March 2021). Data will be made available on request.

## Supplementary Materials

The following supporting information can be downloaded at https://www.mdpi.com/article/10.3390/toxics13040280/s1. Figure S1: B[a]P-induced changes of m6A expression level in HBE-P35 cells; Figure S2: Long-term B[a]P exposure leads to a malignant phenotype in HBE cells; Figure S3: Expression of m6A and YTHDF1 in lung cancer and relationship between YTHDF1 and overall survival time; Figure S4: TMT labelling-based proteomic analysis was performed on YTHDF1 knockdown lung cancer cells and controls; Figure S5: Transfection efficiency; Figure S6: The expression of hsa-miR-139/145-5p. Table S1: Sequence of YTHDF1, CDK6, MAP3K6; Table S2: The expression of differentially expressed proteins in YTHDF1 knockdown cells; Table S3: MAP3K6, CDK6 and PARD6β localization signaling pathways; Table S4: Clinical information and gene expression analysis of tissue microarray; Table S5: Expression analysis of key microRNAs in the GEO database lung cancer population sequencing dataset.
