# Supplementary material for: Effects of Benzo[a]Pyrene Exposure on Lung Cancer: A Mechanistic Study of Epigenetic m6A Levels and YTHDF1
Source: Toxics. 2025 Apr 5;13(4):280. doi: 10.3390/toxics13040280 (PMC12030996; doi:10.3390/toxics13040280)
Supplement: Supplementary file 1 [file toxics-13-00280-s001.zip › toxics-3547548-supplementary.pdf]

Supporting Information

Effects of Benzo[a]Pyrene Exposure on Lung Cancer: A Mechanistic Study of Epigenetic m6A Levels and YTHDF1

Siyi Xu †, Jie Li †, Sheng Yang, Panpan Yang, Yiru Niu, Yiling Ge and Geyu Liang *

Key Laboratory of Environmental Medicine Engineering, Ministry of Education, School of Public Health, Southeast University, Nanjing 210009, China; 15851876530@163.com (S.X.); lijies7@163.com (J.L.); 101300318@seu.edu.cn (S.Y.); yangpanpan199912@163.com (P.Y.); niuyiru666@163.com (Y.N.); geyiling11@163.com (Y.G.)

* Correspondence: lianggeyu@163.com

† These authors contributed equally to this work.

\

catalog

Supporting Information 1
\ 1
Fig. S1 B[a]P-induced changes of m6A expression level in HBE-P35 cells 2
Fig. S2 Long-term B[a]P exposure leads to a malignant phenotype in HBE cells. 3
Fig. S3 Expression of m6A and YTHDF1 in lung cancer and relationship between YTHDF1 and overall survival time. 3
Fig. S4 TMT labelling-based proteomic analysis was performed on YTHDF1 knockdown lung cancer cells and controls. 4
Fig. S5 Transfection efficiency. 4
Fig. S6 The expression of hsa-miR-139/145-5p 5
Table S1 Sequence of YTHDF1, CDK6, MAP3K6 5
Table S2 The expression of differentially expressed proteins in YTHDF1 knockdown cells 6
Table S3 MAP3K6, CDK6 and PARD6β localization signaling pathways 7
Table S4 Clinical information and gene expression analysis of tissue microarray 8
Table S5 Expression analysis of key microRNAs in the GEO database lung cancer population sequencing dataset 9

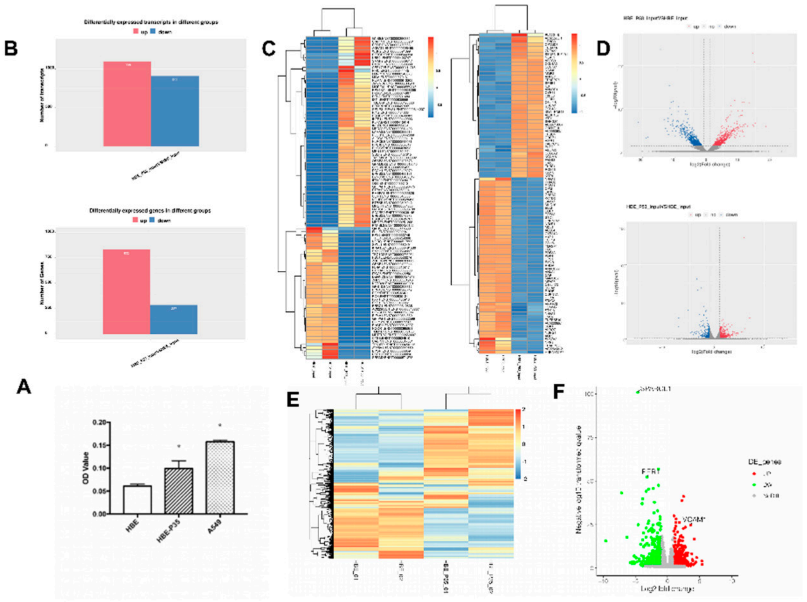

Fig. S1 B[a]P-induced changes of m6A expression level in HBE-P35 cells (A) The level of m6A is highly expressed in HBE-P35 and lung cancer cells; (B,D) Differentially expressed transcripts and genes in HBE-P35 cells; (C) Heat maps of differentially expressed transcripts and genes in HBE-P35 cells; (E) Heat map analysis of differentially expressed genes after B[a]P exposure in MeRIP sequencing analysis; (F) Differentially expressed genes in HBE-P35 cells in MeRIP sequencing analysis.

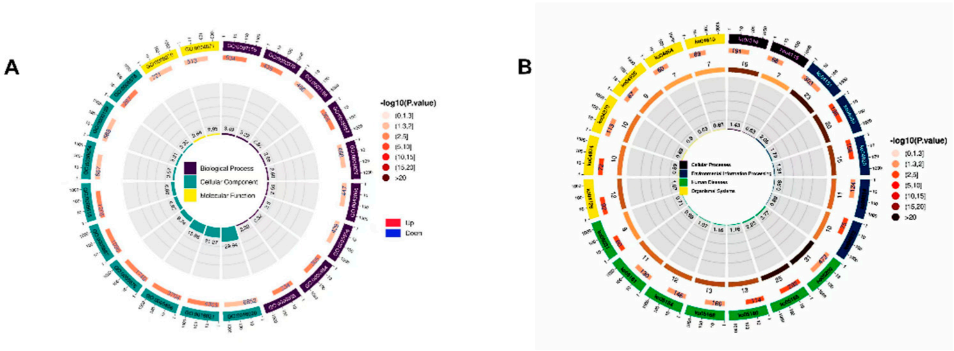

Fig. S2 Long-term B[a]P exposure leads to a malignant phenotype in HBE cells. (A-B) GO and KEGG enrichment analysis.

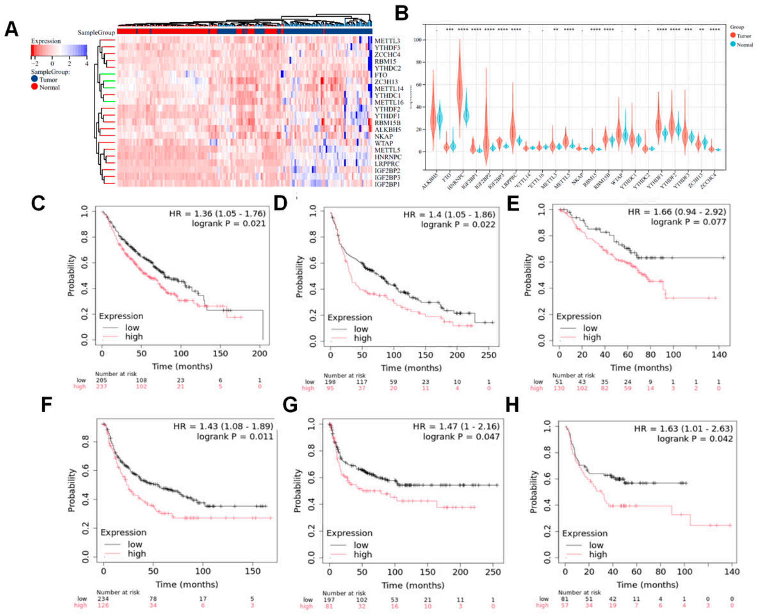

Fig. S3 Expression of m6A and YTHDF1 in lung cancer and relationship between YTHDF1 and overall survival time. (A-B) Expression levels of m6A regulatory factors in TCGA lung cancer dataset. (C) Overall Survival analysis of lung cancer cases with different YTHDF1 expression levels in GSE68465. (D) Overall Survival analysis of lung cancer cases with different YTHDF1 expression levels in GSE30219. (E) Overall Survival analysis of lung cancer cases with different YTHDF1 expression levels in GES50081. (F) Progress Free Survival (PFS) analysis of lung cancer cases with different YTHDF1 expression levels in GES68465. (G) Progress Free Survival (PFS) analysis of lung cancer cases with

different YTHDF1 expression levels in GES30219. (H) Progress Free Survival (PFS) analysis of lung cancer cases with different YTHDF1 expression levels in GES8894.

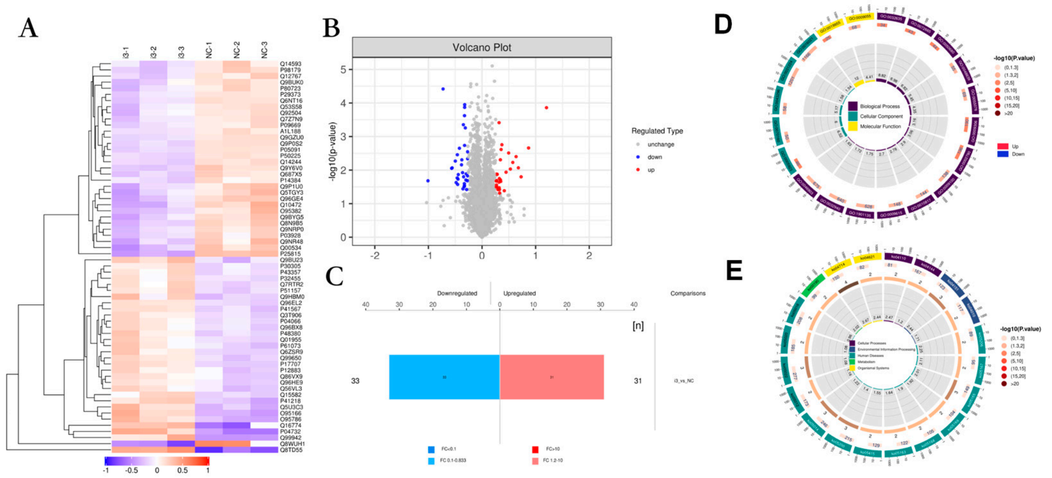

Fig. S4 TMT labelling-based proteomic analysis was performed on YTHDF1 knockdown lung cancer cells and controls. (A) The heat map reveals the difference between the groups with low YTHDF1 and those without low YTHDF1; (B-C) 64 differentially expressed proteins; (D-E) GO and KEGG pathway enrichment analysis using differentially expressed proteins.

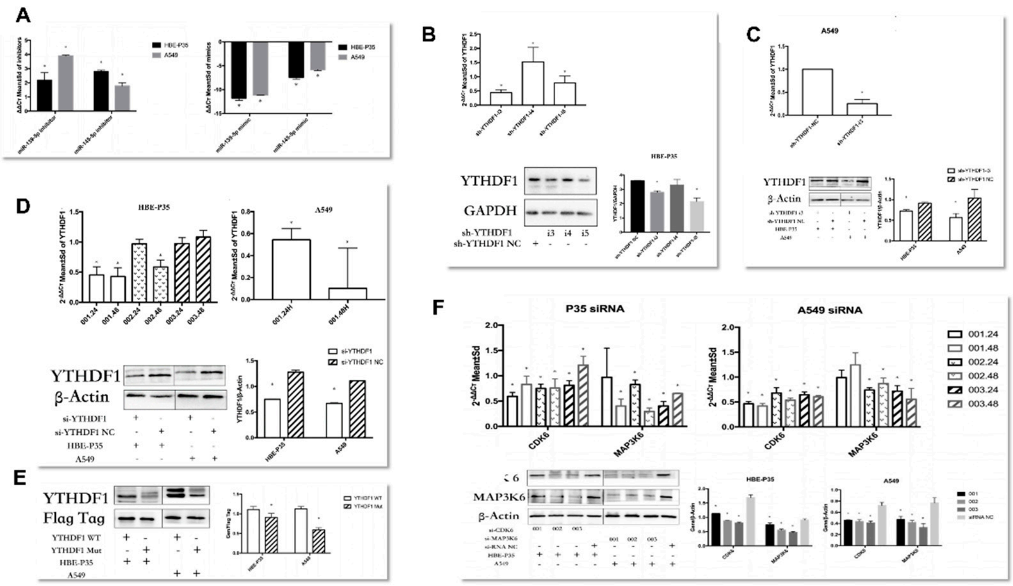

Fig. S5 Transfection efficiency. (A) Transfection efficiency of miR-139/145-5P; (B) The interference efficiency of different sh-YTHDF1 in HBE-P35 cells; (C) The interference efficiency of sh-YTHDF1 in A549 cells; (D) The interference efficiency of si-YTHDF1 in HBE-P35 and A549 cells; (E) The transfection efficiency of YTHDF1-WT/Mut in HBE-P35 and A549 cells; F The interference efficiency of si-CDK6 and si-MAP3K6.

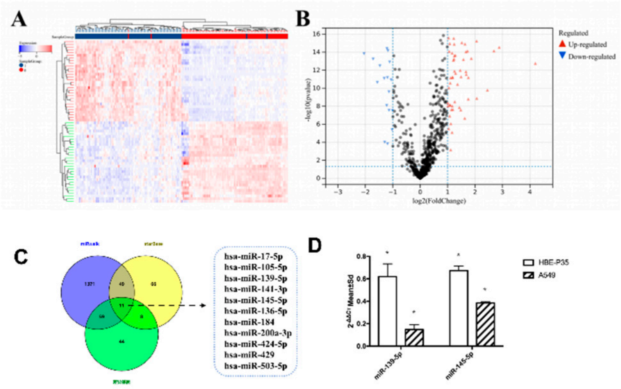

Fig. S6 The expression of hsa-miR-139/145-5p. (A-B) Differential analysis of miroRNAs in paired lung cancer tissue samples in TCGA. (C)TCGA difference analysis results co-screened with miRwalk, starBase prediction results. (D) PCR reveals the expression of hsa-miR-139/145-5p decreased in HBE-P35 cells, and A549 cells compared to HBE cells.

Table S1 Sequence of YTHDF1, CDK6, MAP3K6

Gene	Sequence
YTHDF1-Forward	GGGGACAAGTGGGTCTCAAG
YTHDF1-Reverse	AGGGTGTCGCTGTGAAAGC
GAPDH-Forward	GGGAGCCAAAAGGGTCATCA
GAPDH-Reverse	TGATGGCATGGACTGTGGTC
YTHDF1-i3F	gatccGGCTGGAGAATAACGACAACAtcaagagTGTTGTCGTTATTCTCCAGCCttttt
YTHDF1-i3R	aattaaaaaaGGCTGGAGAATAACGACAACActcttgaTGTTGTCGTTATTCTCCAGCCg
YTHDF1-i4F	gatccgACAGACAGTGTGATGGATGATtcaagagATCATCCATCACACTGTCTGTttttt
YTHDF1-i4R	aattaaaaaACAGACAGTGTGATGGATGATctcttgaATCATCCATCACACTGTCTGTcg
YTHDF1-i5F	gatccgTCACTTCCTCTGAACTGTTACtcaagagGTAACAGTTCAGAGGAAGTGAtttttt
YTHDF1-i5R	aattaaaaaaTCACTTCCTCTGAACTGTTACctcttgaGTAACAGTTCAGAGGAAGTGAcg
st-h-YTHDF1_001	ACGGCAGAGTCGAAACAAA
st-h-YTHDF1_002	CTCCACCCATZZZGCATAA
st-h-YTHDF1_003	GCCGTCCATTGGATTTCCT
st-h-CDK6_001	GCAGAAATGTTTCGTAGAA
st-h- CDK6_002	CAGGCAGGCTTTTCATTCA
st-h- CDK6_003	GTTTGAACATGTCGATCAA
st-h-	GGGACAATGTGCTGATCAA

MAP3K6_001	
st-h-MAP3K6_002	GTGGCCGTATCTACAAGGA
st-h-MAP3K6_003	GCAAGGCTTTTGACGTAGA

Table S2 The expression of differentially expressed proteins in YTHDF1 knockdown cells

Protein Name	Gene Name	FC(i3/NC)	p value
Protein Churchill	CHURC1	0.50	0.02
Protein S100-P	S100P	0.60	0.00
Polypeptide N-acetylgalactosaminyltransferase 1	GALNT1	0.67	0.01
Cyclin-dependent kinase 6	CDK6	0.68	0.01
Centrosomal protein of 95 kDa	CEP95	0.71	0.01
Histone-lysine N-methyltransferase ASH1L	ASH1L	0.72	0.02
Mitogen-activated protein kinase kinase kinase 6	MAP3K6	0.73	0.02
Junction-mediating and -regulatory protein	JMY	0.73	0。00
AT-hook DNA-binding motif-containing protein 1	AHDC1	0.73	0.03
DNA-directed RNA polymerase I subunit RPA12	POLR1H	0.73	0.02
Partitioning defective 6 homolog beta	PARD6β	0.74	0.01
Protein piccolo	PCLO	0.75	0.00
RNA-binding protein 3	RBM3	0.77	0.01
Metalloreductase STEAP4	STEAP4	0.77	0.00
Oligosaccharyltransferase complex subunit OSTC	OSTC	0.78	0.01
ATP synthase protein 8	MT-ATP8	0.78	0.04
Ensconsin	MAP7	0.79	0.00
Transmembrane protein 94	TMEM94	0.79	0.02
Coiled-coil-helix-coiled-coil-helix domain-containing protein 7	CHCHD7	0.79	0.03
Brain acid soluble protein 1	BASP1	0.80	0.03
Zinc finger protein 273	ZNF273	0.80	0.03
Cytochrome c oxidase assembly protein COX16 homolog, mitochondrial	COX16	0.80	0.00
MFS-type transporter SLC18B1	SLC18B1	0.80	0.00
Sulfotransferase 1A1	SULT1A1	0.80	0.00
Aldehyde dehydrogenase, mitochondrial	ALDH2	0.80	0.00
Uncharacterized protein C6orf62	C6orf62	0.81	0.00
Transmembrane protein 177	TMEM177	0.81	0.02
Transmembrane protein 179B	TMEM17B	0.81	0.01
Zinc transporter SLC39A7	SLC39A7	0.82	0.04
Carboxypeptidase M	CPM	0.83	0.02
Cytochrome c oxidase subunit 6C	COX6C	0.83	0.01

NADH dehydrogenase [ubiquinone] 1 alpha subcomplex assembly factor 8	NDUFAF8	0.83	0.00
Cellular retinoic acid-binding protein 2	CRABP2	0.83	0.00
M-phase inducer phosphatase 2	CDC25B	1.20	0.02
Melanoma-associated antigen 3	MAGEA3	1.20	0.03
MOB kinase activator 3A	MOB3A	1.20	0.04
Guanylate-binding protein 1	GBP1	1.21	0.03
Proline-rich protein 11	PRR11	1.21	0.01
Tissue alpha-L-fucosidase	FUCA1	1.21	0.02
Vacuolar fusion protein MON1 homolog A	MON1A	1.21	0.03
N-acetylglucosamine-1-phosphotransferase subunits alpha/beta	GNPTAB	1.22	0.01
Collagen alpha-3(IV) chain	COL4A3	1.22	0.04
Myosin-7	MYH7	1.23	0.04
NLR family CARD domain-containing protein 3	NLRC3	1.24	0.02
28S ribosomal protein S24, mitochondrial	MRPS24	1.25	0。00
Transcription factor RFX3	RFX3	1.25	0.03
Ras-related protein Rab-28	RAB28	1.25	0.02
OCIA domain-containing protein 2	OCIAD2	1.25	0.04
C-X-C chemokine receptor type 4	CXCR4	1.26	0.05
Oncostatin-M-specific receptor subunit beta	OSMR	1.27	0.00
Transforming growth factor-beta-induced protein ig-h3	TGFBI	1.27	0.02
Uncharacterized protein FLJ45252		1.27	0.02
Eukaryotic translation initiation factor 1	EIF1	1.28	0.00
S-adenosylmethionine decarboxylase proenzyme	AMD1	1.29	0.00
Vezatin	VEZT	1.33	0.04
Lipase maturation factor 2	LMF2	1.36	0.01
Myeloid cell nuclear differentiation antigen	MNDA	1.41	0.00
Transmembrane protein 164	TMEM164	1.44	0.01
Antiviral innate immune response receptor RIG-I	DDX58	1.49	0.01
Gamma-aminobutyric acid receptor-associated protein	GABARAP	1.55	0.00
E3 ubiquitin-protein ligase RNF5	RNF5	1.60	0.01
Guanylate kinase	GUK1	1.66	0.02
Metallothionein-1E	MT1E	1.83	0.00
Pleckstrin homology domain-containing family O member 2	PLEKHO2	2.30	0.00

Table S3 MAP3K6, CDK6 and PARD6β localization signaling pathways

Gene Name	Map Name
MAP3K6	MAPK signaling pathway
CDK6	Kaposi sarcoma-associated herpesvirus infection
CDK6	Cell cycle

CDK6	Pancreatic cancer
CDK6	Influenza A
CDK6	Cellular senescence
CDK6	p53 signaling pathway
CDK6	MicroRNAs in cancer
CDK6	Breast cancer
CDK6	Chronic myeloid leukemia
CDK6	Viral carcinogenesis
CDK6	Human cytomegalovirus infection
CDK6	Hepatocellular carcinoma
CDK6	Human papillomavirus infection
CDK6	Measles
CDK6	Hepatitis C
CDK6	PI3K-Akt signaling pathway
CDK6	Pathways in cancer
CDK6	Non-small cell lung cancer
CDK6	Glioma
CDK6	Small cell lung cancer
CDK6	Cushing syndrome
CDK6	Melanoma
CDK6	Epstein-Barr virus infection
PARD-6β	Human papillomavirus infection
PARD-6β	Hippo signaling pathway
PARD-6β	Axon guidance
PARD-6β	Rap1 signaling pathway
PARD-6β	Tight junction
PARD-6β	Endocytosis

Table S4 Clinical information and gene expression analysis of tissue microarray

Features \ Gene		YTHDF1		MAP3K6		CDK6	
		95% confidence interval	*P*	95% confidence interval	*P*	95% confidence interval	*P*
Age		28-72					
Gender	male	[0.478, 0.692]	0.614	[1.020, 1.292]	0.888	[2.330, 2.579]	0.279
	female	[0.363, 0.678]		[0.890, 1.390]		[1.925, 2.660]	
Pathology	LUAD	[0.445, 0.755]	0.889	[0.883, 1.314]	0.320	[2.010, 2.471]	0.034*
	LUSC	[0.465, 0.720]		[1.080, 1.387]		[2.378, 2.667]	
Cancer Stage	stage Ⅰ Ⅱ Ⅲ (resectable)	[0.446, 0.699]	0.719	[0.964, 1.273]	0.260	[2.193, 2.491]	0.621
	stage Ⅲ(unresectable)	[0.448, 0.747]		[1.046, 1.498]		[2.175, 2.638]	
T stage	T1-2	[0.498, 0.730]	0.161	[1.019, 1.308]	0.763	[2.208, 2.474]	0.519
	T3-4	[0.296, 0.629]		[0.940, 1.472]		[2.123, 2.741]	
N stage	N0	[0.468, 0.755]	0.416	[0.961, 1.304]	0.476	[2.137, 2.478]	0.907
	N1-2	[0.403, 0.664]		[1.032, 1.405]		2.240, 2.609]	

Table S5 Expression analysis of key microRNAs in the GEO database lung cancer population sequencing dataset

Database	Time	Country	Sequencing Platform	Group	Sample size	miR-184 FC/*p*	miR-139-5p FC/*p*	miR-145-5p FC/p
GSE15008	2009	China	GPL8176	Lung cancer	120	0.12	-0.06	-1.43
				Control	122	0.34	0.57	<0.01
GSE102287	2017	United States	GPL23871	Lung cancer	91	/	/	-1.17
				Control	88	/	/	<0.01
GSE204951	2022	Italy	GPL19162	Lung cancer	11	2.05	-0.96	-1.46
				Control	12	0.48	<0.01	0.02
GSE72526	2015	Switzerland	GPL20275	Lung cancer	24	0	-0.38	-1.81
				Control	18	1	0.08	<0.01
GSE16025	20109	United States	GPL5106	Lung cancer	61	-0.34	-0.71	-0.48
				Control	10	0.08	<0.01	0.10
GSE19945	2010	Japan	GPL9948	Lung cancer	55	-0.29	-2.66	-2.98
				Control	8	0.31	<0.01	<0.01
GSE169587	2021	Italy	GPL25134	Lung cancer	38	-3.7	-4.16	-3.06
				Control	12	<0.01	<0.01	<0.01
GSE17681	2009	German	GPL9040	Lung cancer	17	2.87	-0.24	-1.69
				Control	19	<0.01	0.76	<0.01
GSE244311	2023	China	GPL23227	Lung cancer	18	-0.96	-1.51	-1.06
				Control	19	0.07	<0.01	<0.01
